# Ostitis

**Published:** 1894-03

**Authors:** Mathew M. Smith

**Affiliations:** Austin


For Texas Medical Journal.

### OSTITIS

BY MATHEW M. SMITH, M. D., AUSTIN.

Read at the meeting of the Austin District Medical Society Dec. 21, 1893.

GENTLEMEN:—The paper I present to-day will be very brief. But I was anxious to have the subject discussed, and had two beautiful bone specimens to exhibit, was why I appeared before you.

Bone being the framework upon which our bodies are built, makes it of great importance; although it is the hardest structure of the human body, yet it is full of blood vessels, lymphatics nerves, etc., and is subject to disease just as the softer parts.

Inflammation of bone is not of uncommon occurrence, and may involve both the soft and more compact portions of bone and its coverings, the periosteum and endosteum. Such conditions are more apt to obtain in early life, owing to the increased blood supply to bone at that age. The bones least covered by tissues are most prone to inflammation.

I shall confine my remarks to ostitis, as the causes, symptoms and treatment in periostitis and endostitis are much the same, though usually lighter in periostitis alone, but as a rule, two or more of these structures are affected simultaneously, owing to their intermediate relations.

In ostitis the bone seems somewhat enlarged, and may gradually lose its density, and become soft and infiltrated with sanguino-plastic material. The capillaries are numerous and full, hence the bone has a reddish appearance. The cellulo-fibrous network is alone the seat of inflammation. The inorganic matter does not undergo change, but may become separated, by the inflammatory changes, from the organic structures. The fibrous matrix of bone undergoes a change, into a fatty transformation. The inorganic material thereby becomes disintegrated, and may be absorbed. By this fatty transformation, there is softening. But the Haversian canals, lacunœ and canaliculi begin to disintegrate and are absorbed, and as a result these cavities show increased size under the microscope, and the compact structure therefore shows a rarified or porous character, and within these spaces the fatty matter and earthy salts and the products of the inflammatory action accumulate; and one kind of deposit results in the hypertrophy and induration of the bone, while the other in an increased softening and disorganization. I present here specimens, one showing the softening, and the other the hardening process by inflammatory action.

If the ostitis be superficial the periosteum is involved, and when deep the endosteum. Ostitis may end in resolution, which is recovery by a gradual disappearance of the morbid symptoms; or terminate with enlarged or indurated bones, or pass into more serious conditions, such as suppuration, ulceration, softening, etc.

Causes of ostitis are manifold, but may be grouped into traumatic and constitutional. Those of the latter are such as syphilis, rheumatism, gout, tuberculosis, etc.

*Symptoms.*—Usually there appears a deep seated wearing pain in the beginning, grows worse, and is very severe and boring in nature, which is increased by motion, pressure, dampness, etc., which becomes worse at night. Enlargement of the bone becomes perceptible, swelling with some pitting of soft parts, and the skin is glossy with an erysipelatous blush. Fever and thirst, dryness of skin. If not relieved, the soft parts suppurate, and rigors, sweats and delirium may even follow. Symptoms of chronic fevers resemble those of the acute form, except they are not so general but of longer duration. The joints may become involved, or the veins carry this abnormal material to the lungs with evil effects.

There is an ostitis deformans described by Sir James Paget, but it is of rare occurrence, and I shall not encroach upon your time with a description of it.

*Treatment.*—General antiphlogistic treatment should be instituted at once. Calomel with opium, perfect rest and especially hot anodyne applications, especially lead water and laudanum. May use blisters or cantharidal collodion or iodine. If you suspect pus, use the knife and remove it, and if necessary to reach it, resort to the trephine. Keep the parts when once opened, dressed with antiseptic precautions. In the chronic form of ostitis it will be necessary to use alteratives, likely counter-irritation will be useful, and such remedies as cod-liver oil, iodine of potassium, etc.; and finally when all these remedies fail you, and your patient continues to grow worse, and the suffering becomes great, and life is in danger, advise amputation as a dernier ressort.

I shall close my paper by referring briefly to two cases operated upon at the hospital, and exhibit the specimens obtained.

Case I. A. S., age about 70 years, negro man. Gives history of good health, no syphiltic or rheumatic history. Says about twenty-five years ago there commenced a small sore on the side of his leg, which he thinks was poisoned by working in the field in the grass, etc. Claims he always had this sore on the leg, but it would improve at times.

When I saw him he suffered severe pain, could not rest for it; had high fever, much thirst, and no appetite. Had been helpless for months. On examination I found the leg much swollen, pitting on pressure, and upon it was a very large chronic ulcer of the leg, with a foul discharge and unhealthy appearance. Closer examination showed a large cavity in the bone from ulceration, and evidences of the tibia and fibula being diseased largely throughout.

After proper local treatment and tonic and hygienic treatment, his health improved some, and I advised an operation, which was done, amputating in the lower third of the thigh. The patient made a good recovery, and I present herewith the specimen obtained.

CASE II. A. M., white man, shepherd, age 60. Gave a history of a chronic ulcer of the leg for twenty-five or thirty years. For many years it would heal up in the winter, and break out in the summer. He could partly follow his vocation, but for the last year or two he was unfit for work, and suffered much pain, and he was so despondent and wretched that he made several attempts at suicide.

Upon examination I found a large chronic ulcer, with unhealthy granulations. The leg was but little swollen, but was hard and dark in color for a large extent, showing the ravages of former attacks, but poorly nourished, and the bone by examination through soft parts, showed evidence of disease. The patient was anxious for an operation, and declared that without it he would seek death. So after a useless attempt to heal the parts, I decided to operate, amputating in the upper third of the leg, during which I had much difficulty in ligating the vessels, owing to a calcareous condition of them. I finally succeeded in tying them high up in the stump. But later the soft parts below the knee became gangrenous, for lack of blood (the anastomosis being very slight above the ligatures). I was therefore compelled to do a secondary amputation (lower third of thigh). The patient made a perfect recovery, and I exhibit the specimen as obtained.

The two cases I report show the results of ostitis in many respects, both acute and chronic, with complications.

